# A Web-Based Survey of Residents' Views on Advocating with Patients for a Healthy Built Environment in Canada

**DOI:** 10.1155/2014/458184

**Published:** 2014-11-11

**Authors:** Matthew Cruickshank, Marcus Law

**Affiliations:** ^1^Department of Family & Community Medicine, Faculty of Medicine, University of Toronto, 840 Coxwell Avenue, Suite 105, Toronto, ON, Canada M4C 5T2; ^2^Faculty of Medicine, University of Toronto, Medical Sciences Building, 1 King's College Circle, Room 2325, Toronto, ON, Canada M5S 1A8

## Abstract

*Purpose*. To determine family medicine residents' perceived knowledge and attitudes towards the built environment and their responsibility for health advocacy and to identify their perceived educational needs and barriers to patient education and advocacy. *Methods*. A web-based survey was conducted in Canada with University of Toronto family medicine residents. Data were analyzed descriptively. *Results*. 93% agreed or strongly agreed that built environment significantly impacts health. 64% thought educating patients on built environment is effective disease prevention; 52% considered this a role of family physicians. 78% reported that advocacy for built environment is effective disease prevention; 56% perceived this to be the family physician's role. 59% reported being knowledgeable to discuss how a patient's environment may affect his/her health; 35% reported being knowledgeable to participate in community discussions on built environment. 78% thought education would help with integration into practice. Inadequate time (92%), knowledge (73%), and remuneration (54%) were barriers. *Conclusions*. While residents perceived value in education and advocacy as disease prevention strategies and acknowledged the importance of a healthy built environment, they did not consider advocacy towards this the family physician's role. Barrier reduction and medical education may contribute to improved advocacy, ultimately improving physical activity levels and patient health outcomes.

## 1. Introduction

Overweight and obesity accompanied by low levels of physical activity are a widespread and growing global problem. Worldwide, the proportion of overweight and obese adults has increased in the last 20 years and continues to increase in children and adolescents in developed and developing countries [1]. In a large US survey, the prevalence of obesity was reported to be 36% in adults [2]. In the UK, obesity in adults and children is amongst the highest prevalence in the developed world [3]. Childhood obesity is increasing in the World Health Organization (WHO) European Region [4], and in Slovenia's capital of Ljubljana overweight and obesity in children have become an epidemic in 20 years spanning from 1991 to 2011 [5]. In Canada, one-quarter of children and youth is overweight or obese, with only 7% attaining the 60 minutes of moderate to vigorous physical activity every day recommended by Canadian guidelines [6]. Similarly, only 15% of Canadian adults attain their recommended level of physical activity, and 61% of Canadian men and 44% of Canadian women are overweight or obese [7]. Lack of physical activity is a risk factor for cardiovascular disease, type 2 diabetes, osteoporosis, depression, anxiety, and some cancers [8], all chronic diseases that are routinely managed by family physicians. Sedentary lifestyle leads to morbidity and mortality for individuals and economic and resource cost for society. Although substantial attention has been paid to promoting healthy lifestyles through individual clinical counselling and mass education programs, activity levels have remained low [9].

Physical activity levels are indirectly influenced by the built environment [10]. One of the most effective means of encouraging physical activity is through urban planning and transportation policies [11]. Urban planning, such as higher residential density, mixed-use zoning, street connectivity, and increased green space, have all been shown to increase physical activity and decrease body mass index [12, 13]. Transportation policies, such as increased public transportation usage, reduced car use, and more infrastructure for walking and cycling, can achieve the same results [12].

Family physicians are well positioned to educate patients on and advocate for a healthy built environment. Given family physicians' long-standing relationships with patients and their role in chronic disease prevention at the individual level, there may be ethical and financial accountability for physicians to take on a larger health advocacy role. The world most recognized and applied physician competency framework, CanMEDS, identified that society believes that a fundamental role of physicians is to act as health advocates at the societal level [14]. With family physicians comprising half of all physicians in Canada, they and their professional associations have great potential to advocate for health considerations in future planning decisions [15].

Family physicians' knowledge and attitudes about their role in educating patients and advocating for healthy built environments and the perceived barriers for doing so are unclear. Therefore, this study sought to measure these constructs in family medicine residents in Canada. As recent graduates of medical school, this population provides a good representation of current medical education.

## 2. Methods

An English-language web-based survey of all 353 family medicine residents in postgraduate year one or year two at the University of Toronto (U of T) was conducted from January to February 2013. There were no exclusion criteria.

Built environment was defined as physical surroundings (i.e., buildings, parks, schools, road systems, and other infrastructures) that we encounter in our daily lives. The survey was designed to address three constructs related to residents educating patients on and advocating for healthy built environment: (1) perceived knowledge, (2) attitudes, and (3) perceived barriers. It was developed based on an extensive literature review of the health impacts of the built environment. Questions to assess perceived knowledge and confidence were developed based on well-researched, high impact topics considered as a reasonable level of knowledge for family physicians. The questionnaire consisted of 21 items rated on a five-point Likert scale (strongly disagree, disagree, neither agree nor disagree, agree, or strongly agree). It also solicited information on relevant demographic information, preferred ways to learn about built environment, and preferred methods to advocate for healthy built environments and educate patients about this topic.

Two family physicians reviewed the questionnaire to determine clarity and face validity. This pilot testing was performed to minimize response error. The questionnaire was subsequently fine-tuned, and questions were added. Ethics approval was obtained from the University of Toronto Research Ethics Board. Consent was implied upon completion of the online survey.

Recruitment emails with a survey link were sent to all potential study participants. The emails stated the purpose of the study, ensured anonymity, and promised a random drawing for gift certificates as an incentive. Using a modified Dillman's method [16], two reminder emails were sent at one-week intervals. Upon the completion of the survey, participants had the opportunity to enter their email address on a separate data collection form for the prize draw.

Data were entered into SPSS 19 for Windows (IBM Corp., Armonk, New York). Descriptive statistics (mean, median, and standard deviation) were calculated to characterize the demographics of the respondents. To simplify the presentation of results, Likert scale responses were trichotomized by collapsing “strongly agree” and “agree” into one category, “strongly disagree” and “disagree” into a second category, and leaving “neither agree nor disagree” as a third category. Frequency distributions and proportions were calculated.

## 3. Results

Three hundred fifty-three individuals received the online survey; 151 surveys were initiated and 140 were completed in full, yielding a response rate of 40%. Seventy-two percent (*n* = 110) of completed surveys were by females, close to the 65% in the sample frame. Nine percent (*n* = 14) of respondents were less than 25 years old, 70% (*n* = 106) were between 25 and 30 years, and 21% were (*n* = 31) older than 30 years. Fifty-four percent (*n* = 82) of respondents were first year residents, and 46% (*n* = 69) were in second year. The respondents completed medical school at 14 of the 17 Canadian schools. While medical curricula may vary across Canada, homogeneity and quality of graduates are guaranteed through independent national organizations responsible for accreditation and certification processes [17]. Fifteen percent (*n* = 23) of respondents were non-Canadian medical graduates.

Residents' attitudes and perceived knowledge and confidence of educating patients on and advocating for healthy built environment are reported in Tables 1 and 2, respectively.

### 3.1. Attitudes

Briefly, 64.1% (*n* = 91) and 77.5% (*n* = 110) of respondents, respectively, indicated that they agreed or strongly agreed with the statements “educating patients on the health impact of the built environment is an effective disease prevention activity” and “advocating for healthy built environments is an effective disease prevention activity.” Approximately half of the respondents agreed or strongly agreed that “it is part of the family physician's role to educate patients on the built environment” and “it is part of the family physician's role to advocate for healthy built environments” (*n* = 74, 52.2%, and *n* = 79, 55.7%, resp.). One-third (*n* = 43, 30.3%) indicated that “it is not the role of family physicians but rather organizations such as the Canadian Medical Association/Ontario Medical Association (CMA/OMA) to educate the public on the health impact of the built environment,” and 38.0% (*n* = 54) felt that “it is not the role of family physicians but rather organizations such as the CMA/OMA to advocate for healthy built environments.” One-third (*n* = 51, 35.9%) perceived that “educating patients on the impact of the built environment is an effective use of family physicians' time.”

### 3.2. Perceived Knowledge

At the individual patient level, slightly more than half of the respondents (*n* = 82, 57.3%) agreed or strongly agreed that they are “well trained to consider a patient's living and work environments as a determinant of their health.” At the population level, only 35.0% (*n* = 50) agreed or strongly agreed that they “feel knowledgeable enough to participate in community discussions on how to improve the health of the local population through changes to the built environment.” Similarly, only one-third (*n* = 43, 30.1%) perceived they were “knowledgeable enough to contribute to a discussion on how urban planning and transportation policies affect health,” while half (*n* = 70, 49.0%) of the respondents disagreed with this statement.

### 3.3. Barriers

Residents' perception of barriers to educate and advocate on this issue by family physicians is reported in Table 3. The majority (*n* = 129, 92.1%) of respondents agreed or strongly agreed that “lack of time” is a barrier, followed by “lack of knowledge” (*n* = 102, 72.8%) and “lack of remuneration” (*n* = 76, 54.3%). Most respondents (*n* = 114, 81.4%) agreed or strongly agreed that they “would benefit from additional education on the health impact of the built environment.”

### 3.4. Educational Preferences

Respondents were asked to select all of their preferred ways to learn about built environment as a determinant of health. Listed in order of frequency, they are lecture (*n* = 94, 67.1%), web module (*n* = 72, 51.4%), and brochure/publication (*n* = 64, 45.7%). Respondents' preferred methods to conduct built environment advocacy and deliver patient education in this area, in descending order, are counselling patients (*n* = 99, 69.7%); having pamphlets, posters, or videos in office (*n* = 95, 66.9%); signing a petition (*n* = 61, 43.0%); participating in government public forums (*n* = 63, 44.4%); writing a letter (*n* = 40, 28.2%); and giving a public lecture (*n* = 34, 23.9%).

## 4. Discussion

This study suggests that while residents perceived value in education and advocacy as disease prevention strategies, they did not agree that it is a role of the family physician to advocate for healthy built environments. Residents are somewhat knowledgeable but lack specific knowledge to participate in larger system level advocacy work. Despite their attitudes, respondents seemed to be open to additional education.

There seemed to be a disconnect between respondents' positive attitudes towards the impact of built environment on health and their lukewarm attitudes towards advocacy as a disease prevention strategy vis-a-vis attitudes towards their role as health advocate (Table 1). One hypothesis is that many of the relationships of built environment to health as identified in the literature are intuitive. For example, most physicians would agree that construction of bike lanes could encourage people to bike to work, subsequently resulting in potential health benefits. However, only half thought that family physicians should have a role to educate patients on and advocate for healthy built environments. This finding is not surprising, given the emphasis of medical education on the medical expert role, but is concerning given the influence that physicians' statements can have on patients' decisions about their care [18, 19]. Other studies that have examined medical residents' attitudes toward health advocacy [20, 21] suggest that although it is generally acknowledged to be part of the physician's social responsibility, residents find few meaningful opportunities to practice advocacy during training, including lack of role models. Improving medical education to address this discordance between advocacy endorsement and lack of engagement may encourage physicians to increase their involvement in meaningful and effective advocacy activities related to this determinant of health. Social responsibility (e.g., advocating for clearer policy) is an important component of community-oriented primary care and seen to be a key role for family physicians [22]. Despite the fact that the role of physicians as health advocate has been long and profusely discussed [14, 23–25], it is not easy to define this role, which may contribute to residents' perceptions of their responsibility to educate patients about and advocate for a healthy built environment.

Interestingly, one-third of survey participants indicated that public education and advocacy for the built environment are roles for medical organizations (Table 1). This attitude may reflect family residents' perceived powerlessness to individually address broader health issues. Residents may also be acknowledging the greater power that physicians have when working together as a collective group. When medical associations or specialists support macro level interventions, it is easier to convince politicians to enact broader change [26]. Thus, educational strategies on how physicians can support the action of the collective may be beneficial but not a total solution as such organizations may not always recognize a priority. A US study, for example, found that despite a national priority to eliminate health disparities, more than half of national physician organizations were doing little to address this problem with relatively few organizations leading most of the efforts [27]. It is unclear to what extent built environment is a priority for such organizations.

A perceived knowledge gap was identified related to advocating for healthy built environments at a broader system level (Table 2). For example, only one-third of respondents reported that their knowledge level was adequate to participate in community discussions on how to improve the health of the local population through changes to the built environment. Most respondents identified that they would benefit from further education on the health impact of the built environment and that this would help them to incorporate this knowledge into their practice (Table 3). These results suggest that current medical education in Canada on this determinant of health is lacking. Other studies also note the knowledge gap [28] and that opportunities for medical trainees to acquire knowledge and skills for health advocacy are lacking [28, 29]. Medical school curriculum has been largely based on biomedical science to the detriment of knowledge acquisition required to achieve and integrate nonmedical expert roles such as communicator and health advocate. Knowledge about the built environment within medical contexts is new for clinicians and educators. Yet, to be able to play an active role in educating patients and advocating for healthier communities, physicians must better understand this topic [30].

While advocacy related to the built environment may be less appropriate at the individual patient level in the office setting, broader ecological interventions and advocacy at the system level are highly relevant. As influential and respected members of society, physicians can have a role in participating in strengthened community action and healthy public policies that support physical activity, safe streets and neighbourhoods, affordable housing, and safe air, soil, and water. As this type of role may require physicians to be more political and may take some doctors outside their comfort zone, educational interventions for physicians would help to support them. Effective teaching of health advocacy competencies in medical school may support future physicians to make this work a true priority.

Lecture and web-based learning modules, identified as preferred formats of continuing education, could be distributed online to all Canadian family physicians at a low cost. E-learning for physicians and medical students is increasingly popular and may offer cost advantages over traditional pedagogical techniques [31, 32]. Increasing physician knowledge on built environment impacts (via web modules) to support preventive or chronic disease management plans would be relevant. However, more creative pedagogy may be needed to fully address skills building for system-level advocacy.

Physicians' prevention and health promotion efforts have been long-studied, and various studies have highlighted barriers to patient education and advocacy [33–36]. In our study, inadequate time to educate patients and advocate for healthy built environment was a perceived barrier for nearly all respondents (Table 3) and only one-third of residents thought that educating patients was an effective use of their time (Table 2). This may reflect a lack of valuing of this issue or lack of clarity around how physician-patient one-on-one encounters will (or will not) create infrastructure or policy changes to support healthy built environments. A broader health advocacy approach may be a more appropriate way to spend time on this issue, and the medical residents may wisely realize this. The fact that residents hold the belief that advocacy or nonclinical work is not appropriately compensated, even before they start earning clinical income, is particularly disconcerting (Table 3). Residents may have become aware of the barrier of inadequate compensation through conversations with practicing physicians. This barrier may not be specific to advocating for healthy built environment but, rather, may extend to the overall lack of compensation for nonclinical work. In Canada, the funding models have remained relatively consistent over time for preventive care activities. This link between remuneration and preventive care has been reported elsewhere in the literature [33, 36, 37].

The next step will be to expand this research beyond medical residents to include currently practicing Canadian family physicians.

Furthermore, targeted medical education interventions that foster a sense of responsibility related to health advocacy are indicated. By the time residents become doctors, if they have not already acknowledged the importance of their role in advocacy for a healthy built environment, it is possible that they may never engage in broader advocacy efforts.

The study has a few limitations. The 40% survey response rate suggests a degree of nonresponse error. Physician surveys are often characterized by low response rates [38]. While a high response rate is sought after, research evidence indicates that physician surveys are somewhat more resilient to the effects of nonresponse compared with general population surveys. Response bias may be less of a concern in this case, as most nonresponse studies have found no or only minimal amounts of response bias [39–41]. Furthermore, the distribution of respondents by sex was similar between the sample population and the respondents, suggesting a satisfactory level of representation. A second limitation is that the survey questions were not validated as to whether they addressed the study constructs. In an effort to reduce this error, the questions were developed with a thorough literature review and multiple iterations. Finally, response error was minimized by cognitive interviewing and multiple pilot surveys to examine comprehensibility.

## 5. Conclusions

Effective urban planning (i.e., urban design, land use) and transportation policies can impact the health of Canadians, and family physicians have the potential to be both educators and advocates for healthy built environments. This survey revealed ambivalent attitudes among residents towards a physician's role in education and advocacy for a healthy built environment. Family medicine residents acknowledged that the built environment significantly impacts health but were divided on whether family physicians have a role in advocacy. They perceived inadequate time, knowledge, and, to a lesser extent, remuneration as barriers to education and advocacy related to the built environment. Family medicine residents reported having some knowledge of the built environment but would benefit from further education, particularly on ways to engage in system-level advocacy. Some residents wisely acknowledged that professional organizations have a role to play in improvements to the built environment, as such advocacy may be best addressed through collective action.

This study supports the idea that implementing physician support and educational interventions to address attitudes and skills deficits related to patient education and system-level advocacy for the built environment would be a step towards effective disease prevention, such as improving physical activity levels.

## Supplementary Material

The Supplementary Material provided is the survey used in our study. The survey instrument was a questionnaire to assess residents' perceived knowledge and confidence towards advocating for healthy built environments. The questionnaire consisted of 21 items rated on a five-point Likert scale (strongly disagree, disagree, neither agree nor disagree, agree, or strongly agree).

## Figures and Tables

**Table 1:** Family medicine residents' attitudes towards educating patients on and advocating for healthy built environment.

Statement	Strongly disagree or disagree *N* (%)	Not sure *N* (%)	Agree or strongly agree *N* (%)
The built environment has a significant effect on the health of the Canadian population	1 (0.7)	9 (6.3)	132 (92.9)

Educating patients on the health impact of the built environment is an effective disease prevention activity	12 (8.4)	39 (27.5)	91 (64.1)

Preventive care counseling is an important part of my practice	1 (0.7)	3 (2.1)	136 (97.2)

It is part of the family physicians' role to educate patients on the built environment	17 (12.0)	51 (35.9)	74 (52.2)

Educating patients on the health impact of the built environment is an effective use of family physicians time	40 (28.2)	51 (35.9)	51 (35.9)

It is not the role of family physicians but rather organizations such as the CMA/OMA to educate the public on the health impact of the built environment	39 (27.5)	60 (42.3)	43 (30.3)

Advocating for healthy built environments is an effective disease prevention activity	4 (2.8)	28 (19.7)	110 (77.5)

Preventive care advocacy is an important part of my role as a family physician	0 (0)	4 (2.9)	136 (97.1)

It is part of the family physicians' role to advocate for healthy built environments	10 (7.0)	53 (37.3)	79 (55.7)

It is not the role of family physicians but rather organizations such as the CMA/OMA to advocate for healthy built environments	35 (24.6)	53 (37.3)	54 (38.0)

**Table 2:** Family medicine residents' knowledge and confidence educating patients on and advocating for healthy built environment.

Statement	Strongly disagree or disagree *N* (%)	Not sure *N* (%)	Agree or strongly agree *N* (%)
I am well trained to consider a patient's living and work environments as a determinant of their health	22 (15.4)	39 (27.3)	82 (57.3)

I feel knowledgeable enough to discuss with a patient how their living and work environment may be affecting their health	20 (14.0)	39 (27.3)	84 (58.7)

I feel knowledgeable enough to discuss with patients how their transportation choices may be affecting their health	24 (16.8)	47 (32.9)	72 (50.4)

I am well trained to make suggestions to patients on how they can improve their physical and mental health by making changes to their living and/or working environment(s)	31 (21.7)	37 (25.9)	75 (52.5)

I feel knowledgeable enough to participate in community discussions on how to improve the health of the local population through changes to the built environment	55 (38.5)	38 (26.6)	50 (35.0)

I feel knowledgeable enough to contribute to a discussion on how urban planning and transportation policies affect health	70 (49.0)	30 (21.0)	43 (30.1)

**Table 3:** Family medicine residents' perceived barriers to educating patients on and advocating for healthy built environment.

Statement	Strongly disagree or disagree *N* (%)	Not sure *N* (%)	Agree or strongly agree *N* (%)
Lack of time is a barrier in educating on and advocating for healthy built environments	4 (2.9)	7 (5.0)	129 (92.1)

Lack of remuneration is a barrier in educating on and advocating for healthy built environments	21 (15.0)	43 (30.7)	76 (54.3)

Lack of knowledge is a barrier in educating on and advocating for healthy built environments	16 (11.4)	22 (15.7)	102 (72.8)

I would benefit from additional education on the health impact of the built environment	7 (5.0)	19 (13.6)	114 (81.4)

Additional education on the health impact of the built environment would help me to incorporate it into my work life	9 (6.4)	22 (15.7)	109 (77.8)
