# Supplementary material for: A Web-Based Survey of Residents' Views on Advocating with Patients for a Healthy Built Environment in Canada
Source: Int J Family Med. 2014 Nov 11;2014:458184. doi: 10.1155/2014/458184 (PMC4243584; doi:10.1155/2014/458184)
Supplement: Supplementary file 1 — The Supplementary Material provided is the survey used in our study. The survey instrument was a questionnaire to assess residents' perceived knowledge and confidence towards advocating for healthy built environments. The questionnaire consisted of 21 items rated on a five-point Likert scale (strongly disagree, disagree, neither agree nor disagree, agree, or strongly agree). [file 458184.f1.pdf]

Appendix A: Questionnaire to assess residents' perceived knowledge and confidence towards advocating for healthy built environment

Survey Instrument

Demographics

Your gender is: ☐ **Male** ☐ **Female**

How old are you? _____ **years old**

Your current residency year ☐ **PGY1** ☐ **PGY2**

Where did you complete your undergraduate medical degree? ________________

Section 1: Knowledge of the health impact of the built environment

The term 'built environment' in this survey refers to our physical surroundings which includes the buildings, parks, schools, road systems, and other infrastructure that we encounter in our daily lives.

To what extent do you agree with the following statements?

	Strongly Agree	Agree	Neither Agree Nor Disagree	Disagree	Strongly Disagree
1. I am well trained to consider a patient's living and work environments as a determinant of their health	☐	☐	☐	☐	☐
2. I feel knowledgeable enough to discuss with a patient how their living and work environment may be affecting their health	☐	☐	☐	☐	☐
3. I feel knowledgeable enough to discuss with a patient how their transportation choices may be affecting their health	☐	☐	☐	☐	☐
4. I am well trained to make suggestions to patients on how they can improve their physical and mental health	☐	☐	☐	☐	☐

by making changes to their living and/or work environment(s)					
5. I feel knowledgeable enough to participate in community discussions on how to improve the health of the local population through changes to the built environment	☐	☐	☐	☐	☐
6. I feel knowledgeable enough to contribute to a discussion on how urban planning and transportation policies affect health	☐	☐	☐	☐	☐

Section 2: Attitudes - The advocacy role of the family physician

The term 'built environment' in this survey refers to our physical surroundings which includes the buildings, parks, schools, road systems, and other infrastructure that we encounter in our daily lives.

To what extent do you agree with the following statements?

	Strongly Agree	Agree	Neither Agree Nor Disagree	Disagree	Strongly Disagree
7. The built environment has a significant effect on the health of the Canadian population	☐	☐	☐	☐	☐
8. Educating patients on the health impact of the built environment is an effective disease prevention activity	☐	☐	☐	☐	☐
9. It is part of the family physician's role to educate patients on the health impact of the built environment	☐	☐	☐	☐	☐
10. Educating patients on the health impact of the built environment is an effective use of family physicians' time	☐	☐	☐	☐	☐
11. It is not the role of	☐	☐	☐	☐	☐

family physicians but rather organizations such as the CMA/OMA to educate the public on the health impact of the built environment					
12. Advocating for healthy built environments is an effective disease prevention activity	☐	☐	☐	☐	☐
13. It is part of the family physician’s role to advocate for healthy built environments	☐	☐	☐	☐	☐
14. It is not the role of family physicians but rather organizations such as the CMA/OMA to advocate for healthy built environments	☐	☐	☐	☐	☐

15. Your preferred means of built environment education and advocacy (choose all that apply):

Counsel patients ☐
Have pamphlets/posters/video in office ☐
Sign a petition ☐
Participate in government public forums ☐
Write a letter ☐
Give a public lecture ☐
None of the above ☐

<u>Section 3: Attitudes - Perceptions of barriers</u>

The term ‘built environment’ in this survey refers to our physical surroundings which includes the buildings, parks, schools, road systems, and other infrastructure that we encounter in our daily lives.

To what extent do you agree with the following statements?

	Strongly Agree	Agree	Neither Agree Nor Disagree	Disagree	Strongly Disagree
16. Preventative care counseling is an important part of my practice	☐	☐	☐	☐	☐
17. Preventative care advocacy is an important part of my role as a family physician	☐	☐	☐	☐	☐

18. Lack of time is a barrier in educating on and advocating for healthy built environments	☐	☐	☐	☐	☐
19. Lack of remuneration is a barrier in educating on and advocating for healthy built environments	☐	☐	☐	☐	☐
20. Lack of knowledge is a barrier in educating on and advocating for healthy built environments	☐	☐	☐	☐	☐
21. I would benefit from additional education on the health impact of the built environment	☐	☐	☐	☐	☐
22. Additional education on the health impact of the built environment would help me to incorporate it into my work life	☐	☐	☐	☐	☐

23. Your preferred form(s) of education are (choose all that apply):

Brochure/publication ☐
Web module ☐
Lecture ☐
None of the above ☐
